# Phage Resistance Evolution Induces the Sensitivity of Specific Antibiotics in Pseudomonas aeruginosa PAO1

**DOI:** 10.1128/spectrum.01356-22

**Published:** 2022-08-16

**Authors:** Guanhua Xuan, Hong Lin, Jiuna Kong, Jingxue Wang

**Affiliations:** a Food Safety Laboratory, College of Food Science and Engineering, Ocean University of China, Qingdao, China; Agriculture and Agriculture-Food Canada

**Keywords:** *Pseudomonas aeruginosa*, phage therapy, phage-resistant, antibiotic sensitivity, trade-off

## Abstract

Bacteria frequently encounter selection by both phages and antibiotics. However, our knowledge on the evolutionary interactions between phages and antibiotics are still limited. Here, we characterized a phage-resistant Pseudomonas aeruginosa variant PAO1-R1 that shows increased sensitivity to gentamicin and polymyxin B. Using whole genome sequencing, significant genome differences were observed between the reference P. aeruginosa PAO1 and PAO1-R1. Compared to PAO1, 64 gene-encoding proteins with nonsynonymous single nucleotide polymorphisms (SNPs) and 31 genes with insertion/deletion (indel) mutations were found in PAO1-R1. We observed a significant reduction in phage adsorption rate for both phage vB_Pae_QDWS and vB_Pae_W3 against PAO1-R1 and proposed that disruption of phage adsorption is likely the main cause for evolving resistance. Because the majority of spontaneous mutations are closely related to membrane components, alterations in the cell envelope may explain the antibiotic-sensitive phenotype of PAO1-R1. Collectively, we demonstrate that the evolution of phage resistance comes with fitness defects resulting in antibiotic sensitization. Our finding provides new insights into the evolutionary interactions between resistance to the phage and sensitivity to antibiotics, which may have implications for the future clinical use of steering in phage therapies.

**IMPORTANCE** Bacteria frequently encounter the selection pressure from both antibiotics and lytic phages. Little is known about the evolutionary interactions between antibiotics and phages. Our study provides new insights into the trade-off mechanism between resistance to the phage and sensitivity to antibiotics. This evolutionary trade-off is not dependent on the outer membrane proteins (OMPs) of the multidrug efflux pumps. The disruption of phage adsorption that induced phage resistance and the changes in structure or composition of membranes are presumably one of the major causes for antibiotic sensitivity. Our finding may fill some gaps in the field of phage-host interplay and have implications for the future clinical use of steering in phage therapies.

## INTRODUCTION

Pseudomonas aeruginosa (P. aeruginosa) is a Gram-negative bacterium with a large genome (5.5–7 Mbp), which allows for great metabolic versatility and high adaptability to environmental changes (1). As an important foodborne opportunistic pathogen, P. aeruginosa also acquired multidrug resistance (MDR), which is considered as one of the biggest threats to public health (2). Though novel classes of antibiotics have been discovered, very few antibiotics reach the clinical application due to safety concern. Therefore, there is an urgent need to develop effective novel approaches for treatment of infections induced by this pathogen (3, 4).

The bacteriophage (phage) therapy is a promising alternative to conventional antibiotics for treating MDR bacterial infections (5), but it still faces some challenging issues, e.g., bacteria readily evolve resistance to phage infection. Phage-resistant bacteria can result from several mechanisms, such as restriction-modification (6), abortive infection (7), CRISPR/Cas9 mechanisms (8), and mutations in phage receptor sites (9). However, phage resistance is often accompanied by a reduction in fitness traits, such as reducing growth rates and decreasing virulence (10). More detailed understanding of the evolutionary trade-offs between increased phage resistance and decreased fitness traits is critical for phage therapy in treating bacterial infections.

Potential evolutionary trade-offs between phage resistance and antibiotic sensitivity have been previously identified (11). These evolutionary trade-offs were reported to be closely associated with the cell membrane proteins of multidrug efflux pumps. For example, the outer membrane protein (OMP) components TolC and OprM are frequently the receptors for phages and are also the essential components for multidrug efflux pumps, whereby the evolution of bacterial resistance to phage attack could change the efflux pump mechanism, causing increased antibiotic sensitivity (11, 12). Although, in general, the evolutionary interactions between antibiotics and phages remain unclear.

Here, we isolated and characterized a phage-resistant P. aeruginosa derivative PAO1-R1. We verified the phage receptor-mediated reduction of adsorption rate is the important strategy for PAO1-R1 to defend against phage infection. In addition, phage resistance induced the fitness costs in P. aeruginosa, which making *P. aeruginosa* more susceptibility to both gentamicin and polymyxin B. We attribute the antibiotic sensitivity largely to changes in the structure of the cell membrane. We proposed the trade-off mechanism between phage resistance and antibiotic sensitivity did not rely on the OMPs of the multidrug efflux pumps that were previously reported. Our new discovery will fill some gaps in the field of phage-host interplay.

## RESULTS

### Phage-resistant P. aeruginosa variant characterization.

**FIG 1:**
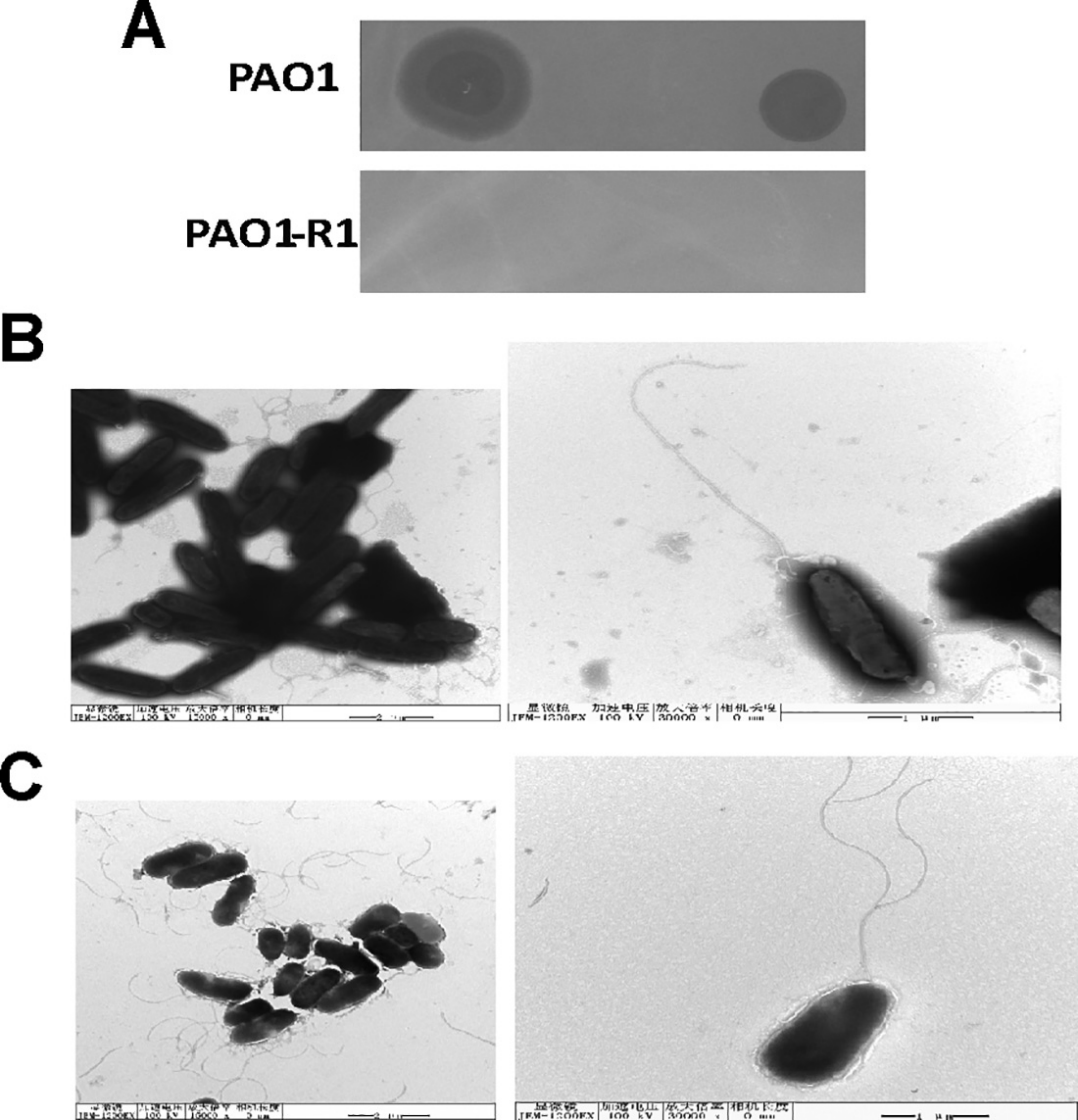
Characterization of the phage-resistant strain PAO1-R1. (A) Spot test of phages vB_Pae_QDWS and vB_Pae_W3 at high titers (10^6^ PFU/mL) when plated on PAO1 and PAO1-R1. Electron micrograph of (B) P. aeruginosa PAO1 and (C) PAO1-R1 cells.

When Pseudomonas aeruginosa phages vB_Pae_QDWS and vB_Pae_W3 were assayed on PAO1 strains, a clear plaque formed. However, there were no lytic activities for the two phages when plated on PAO1-R1 (Fig. 1A). Results of the growth curves of PAO1 and PAO1-R1 infected with phages also showed PAO1-R1 is resistant to both vB_Pae_QDWS and vB_Pae_W3 (Fig. S1). Transmission electron microscopy (TEM) was performed on PAO1 and PAO1-R1. Unlike PAO1 (Fig. 1B), PAO1-R1 decreased the cell length and exhibited roundish (coccobacilli) forms (Fig. 1C). The overall cell morphology changes happened on the phage-resistant PAO1-R1 strains.

**FIG 2:**
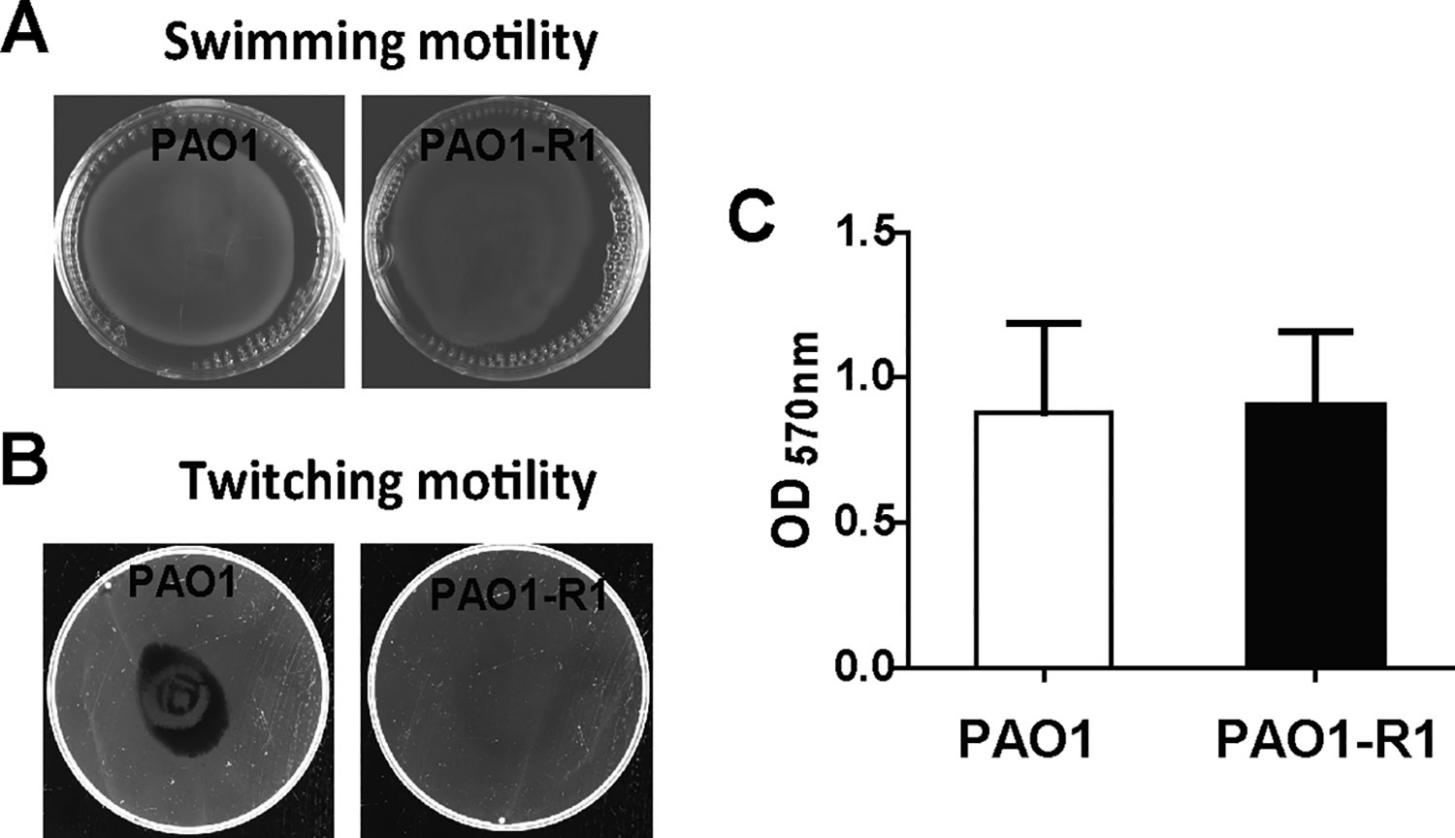
Twitching and biofilm phenotypes for wild-type PAO1 and PAO1-R1. Swimming agar (A) and twitching agar (B) for PAO1 and PAO1-R1 strains. (C) Quantification of biofilm biomass. Error bars denote standard deviations among triplicate samples.

The motility system and biofilm formation are related to virulence (18), which is necessary for P. aeruginosa to adjust to diverse environmental conditions. Results of motility assays showed no difference in swimming motility and biofilm production between PAO1 and PAO1-R1; however, there was a definite loss of twitching motility in PAO1-R1compared to PAO1 (Fig. 2).

### Assessment of the alteration in phage adsorption rates of PAO1-R1.

**FIG 3:**
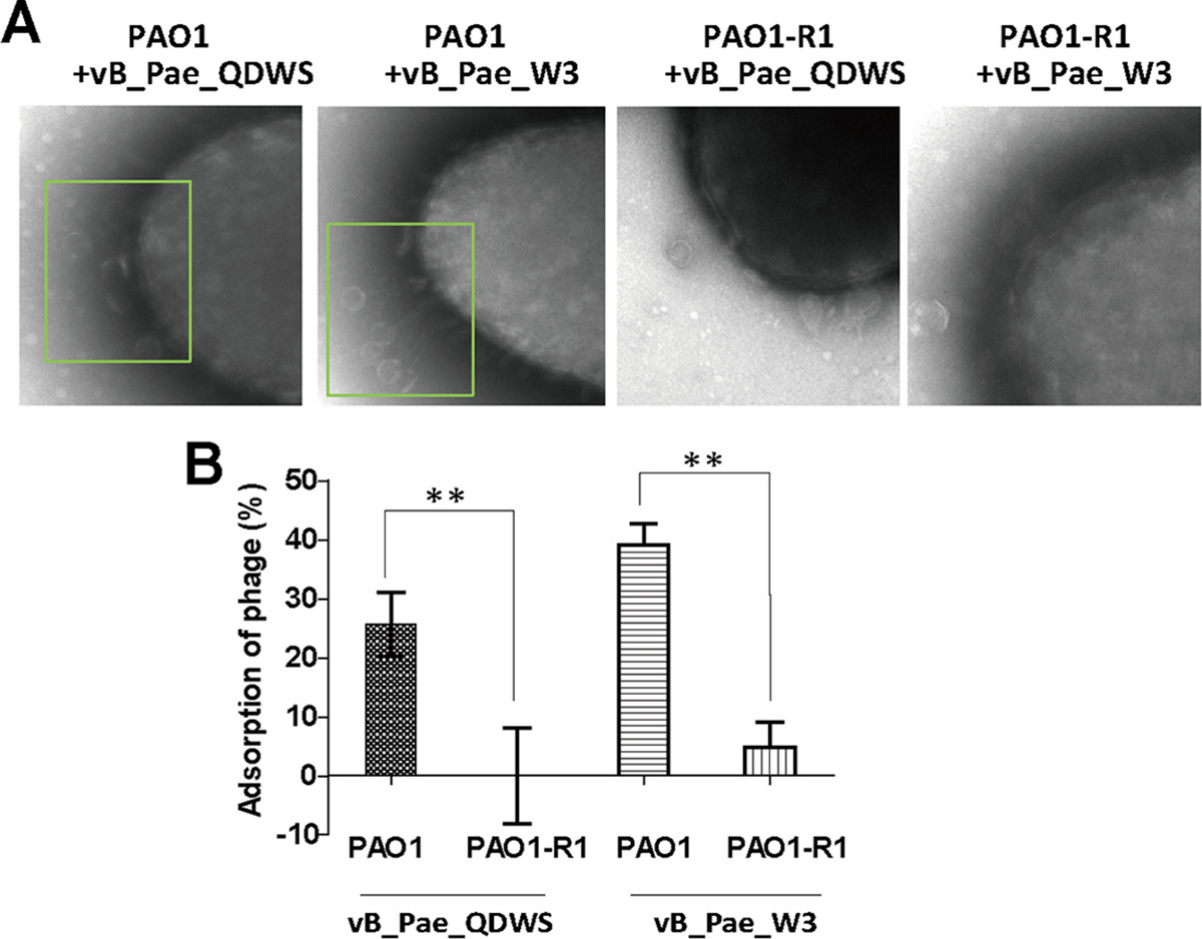
Phage adsorption assay. (A) PAO1 and PAO1-R1 strains were mixed with phages vB_Pae_QDWS and vB_Pae_W3 at an MOI of 100 for 5 min and subjected to TEM analysis. (B) Adsorption assays of phages vB_Pae_QDWS and vB_Pae_W3 for PAO1 and PAO1-R1 strains. The values were the averages of three measures with standard deviation. T tests were performed to calculate the *P*-values, and double asterisks indicate statistically significant difference (**, *P* < 0.01).

It has been reported that the twitching motility of P. aeruginosa depends on the integrity of the T4P (Type IV pilus) (19, 20). Both phages vB_Pae_QDWS and vB_Pae_W3 could recognize T4P as their receptor sites (21). To investigate whether the alteration in the T4P receptor affects antiphage infection of PAO1-R1, we performed an adsorption assay. TEM results showed that almost no phages attached to the surface of the PAO1-R1 (Fig. 3A). By adsorption rate assay, we found that PAO1-R1 significantly reduced the adsorption of both phage vB_Pae_QDWS and vB_Pae_W3 compared with PAO1 (Fig. 3B). Hence, we propose that the impairment of T4P-mediated phage adsorption was the main cause of PAO1-R1 phage resistance.

### PAO1-R1 restored gentamicin and polymyxin B sensitivity.

**FIG 4:**
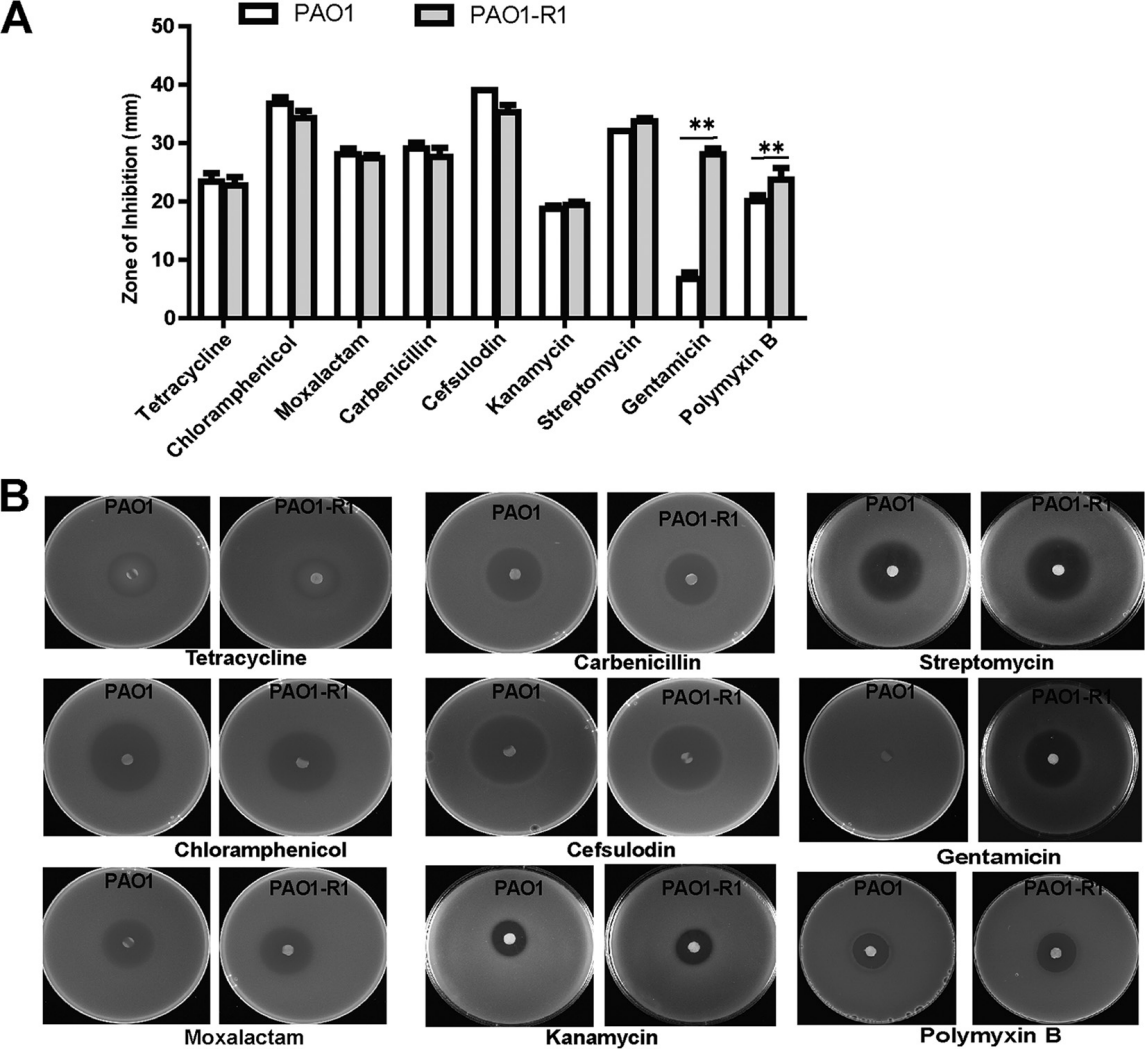
Gentamicin and polymyxin B susceptibility. (A) Nine antibiotics, including tetracycline, moxalactam, cefsulodin, chloramphenicol, carbenicillin, kanamycin, streptomycin, gentamicin, and polymyxin B were used for resistance assay. (B) Filter paper discs containing several classes of antibiotics were placed onto LB agar plates containing two P. aeruginosa strains, the wild-type PAO1 and PAO1-R1. The plates were cultured at 37°C overnight and the diameters of the inhibition zone were measured. The values were the averages of three measures with standard deviation. T tests were performed to calculate the *P*-values, and double asterisks indicate statistically significant difference (**, *P* < 0.01).

Nine antibiotics were selected for the antibiotic sensitivity assays in P. aeruginosa PAO1 and PAO1-R1. In disk diffusion assays, no significant difference in tetracycline, moxalactam, cefsulodin, chloramphenicol, carbenicillin, streptomycin, or kanamycin sensitivity between the wild type PAO1 and PAO1-R1 was found (Fig. 4A). However, the phage-resistant strain PAO1-R1 increased sensitivity to gentamicin and polymyxin B relative to PAO1 (Fig. 4).

The MIC results also revealed that the inhibitory antibiotic concentrations of PAO1-R1 were equivalent or lower than PAO1. For PAO1-R1, we observed a 2-fold reduction in the MIC of gentamicin and polymyxin B (Table 1), which is consistent with the results of the disk diffusion assay (Fig. 4). No obvious differences were found in the MIC of the other seven antibiotics for PAO1 and PAO1-R1. These results suggest that the acquisition of phage resistance caused a fitness defect that manifests as enhanced sensitivity to certain antibiotics.

**TABLE 1:** Determination of MIC for PAO1 and phage-resistant isolate PAO1-R1^*a*^

Antibiotics	MIC (μg/mL)		IC breakpoints^*b*^			Comments
	PAO1	PAO1-R1	S	I	R	
Tetracycline	8	8	≤4	8	≥4	Standard for reference *Enterobacteriaceae* breakpoints
Moxalactam	256	256	≤8	16–32	≥64	
Cefsulodin	1	1	ND	ND	ND	
Chloramphenicol	32	32	≤8	16	≥32	
Carbenicillin	128	128	ND	ND	ND	
Kanamycin	64	64	≤16	32	≥64	
Streptomycin	4	4	ND	ND	ND	
Gentamicin	2	1	≤4	8	≥16	*P. aeruginosa* breakpoints
Polymyxin B	16	8	≤2	4	≥8	

a P. aeruginosa strains were deemed susceptible or resistant to antibiotics according to the CLSI P. aeruginosa breakpoints.

*^b^*ND, not determined; R, resistant; I, immediate; S, susceptible.

### Comparative genomics analysis.

Comparative genomic analysis was performed to reveal the detailed genetic differences between PAO1 and PAO1-R1. Analysis of genetic variation across the whole genomes showed that a total of 166 SNPs were identified in PAO1-R1, and 64 gene-encoding proteins showed amino acid changes caused by nonsynonymous SNPs (Table S1). These genes were assigned to 5 clusters by Gene Ontology (GO) analysis using the Database for Annotation, Visualization and Integrated Discovery (DAVID), with 41 genes clustered (Table 2). The majority of genes with the SNP variants were predicted to be membrane proteins and transcription regulators. In addition, 32 indel mutations in 31 genes occurred in PAO1-R1 using PAO1 as a reference (Table S2). The mutation in 6 genes (PA0683, *napA*, PA1327, *mtnC*, PA2492, and *amiE*) showed high impact on protein activity, which was predicted by snpEFF software (22).

**TABLE 2:** Classification of SNPs found in PAO1-R1-associated ORFS

Function cluster	Annotation	No. of genes	Gene name
Cluster 1	Membrane	20	PA2409, PA2541, PA1923, *lepA*, *fliF*, *kefB*, PA0801, PA0847, PA4394, PA1108, PA1361, *pyoS5*, *pntB*, PA3522, PA1611, *cysW*, *tolA*, *pcrD*, *mtr*, *pilQ*
Cluster 2	Plasma membrane	9	PA2409, *lepA*, *fliF*, *kefB*, PA1361, *phoU*, *pntB*, *cysW*, *tolA*
Cluster 3	Transcription regulation	8	*putA*, *hutC*, PA0056, PA0159, PA0839, PA2383, PA4341, *gltR*
Cluster 4	CheY-like superfamily	3	PA1459, PA1611, *gltR*
Cluster 5	ATP binding protein	6	*cysS*, *proB*, PA0716, *liuD*, PA1611, *pprA*
Not clustered		23	

## DISCUSSION

We present evidence of a phage-resistant P. aeruginosa variant PAO1-R1 against phages vB_Pae_QDWS and vB_Pae_W3, that shows increased sensitivity to gentamicin and polymyxin B. Phage selection produces an evolutionary trade-off in P. aeruginosa. To gain insight into the coevolution mechanism, we performed the morphological characterization, adsorption rate determination, and comparative genomic analysis. Our results demonstrated that spontaneous mutations were likely the main mechanisms driving phage-bacterial coevolution.

Preventing phage adsorption is a common mechanism that bacteria evolved to defend against invading phages (23). T4P are not only the virulence factors for some pathogens (24–26), but also the receptors for many P. aeruginosa phages (27, 28). Specific amino acid substitutions in T4P synthesis-related genes or specific modifications on T4P would significantly decrease the susceptibility of strains to phage infection (29). This is consistent with a previous report that the majority of detected phage-resistant mutant strains were shown to resist phage infection via blocking phage adsorption (30). Our data also suggest the reduction of the T4P-mediated adsorption rate is the major strategy for the resistant mutant PAO1-R1 to defend against phage vB_Pae_QDWS and vB_Pae_W3 (Fig. 3). The T4P comprise a cell envelope-spanning multiprotein complex, and PilQ (fimbrial assembly protein, encoded by *pilQ*) is required for T4P biosynthesis in P. aeruginosa (31). It has been reported that *pilQ* mutants lack the spreading colony morphology characteristic of twitching motility and are resistant to the pilus-specific phages (32). The results of comparative genomics showed that two point mutations (Lys693Glu and Val642Ile) were found in the *pilQ* gene (Table S1), which are the only gene missense mutations related to pilus synthesis that were found in PAO1-R1. As expected, PAO1-R1 also exhibited the absence of twitching motility (Fig. 2B) and a phage resistant phenotype (Fig. 1A). However, it is unclear whether Lys693 and Val642 mutations of PilQ directly affect T4P function and then influence phage resistance. Unfortunately, we have not been able to obtain *pilQ* mutants or a *pilQ* overexpression strain, which also implies that the key roles of PilQ and its expression may be strictly regulated, although the *pilQ* gene is considered to be one of the most frequent mutations in T4P genes (33). Helm et al. (34) generated 19 unique *pilQ* missense mutations and found that they have diverse effects on pilus function in Neisseria gonorrhoeae, which further indicates the role of PilQ in the biology of the T4P. Additional analysis of the Lys693 and Val642 mutants is necessary in the future to support our hypothesis that the *pilQ* missense mutations influence phage resistance.

No obvious growth difference was observed between PAO1 and PAO1-R1 (Fig. S1). However, the significant increase in the sensitivity to certain antibiotics occurred in PAO1-R1, which is similar to the previous report (35). PAO1-R1 displayed more susceptible to gentamicin and polymyxin B compared to PAO1 (Fig. 4). Gentamicin acts as a ribosome inhibitor that could cause protein mistranslation and bacterial death (36). Galbraith et al. (37) report that the structural alterations in the cell envelope were correlated with gentamicin sensitivity. This is also consistent with one observation that changes in outer membrane proteins and lipopolysaccharide in cells of strains of Pseudomonas stutzeri showed alterations in gentamicin resistance (38). For polymyxin B, it could combine its diaminobutyric acid residues (DAB) to the lipid A, leading to the changes in cell integrity and permeability (39, 40). Both gentamicin and polymyxin B could display lethal effects on bacterial cells through perturbation of the cell surface (41, 42). PAO1-R1 had obvious morphology changes (Fig. 1C) that closely related to membrane structure changes (43, 44), though almost no differences in the membrane permeability between PAO1 and PAO1-R1 were found by 1-N-phenylnaphthylamine (NPN) assay (Fig. S2). Combined with our findings, we speculate that alterations in the cell envelope are likely one of the important reasons for antibiotic sensitivity. Further study is helpful to clarify why PAO1-R1 showed sensitive to gentamicin and polymyxin B.

This evolutionary trade-off phenomenon we proposed is quite different from the multidrug efflux systems-mediated mechanism, since the key receptor for phages vB_Pae_QDWS and vB_Pae_W3 was determined to be T4P (Fig. 3), rather than the OMPs component. Our data demonstrated that spontaneous mutation is the main driving force for the coevolution of bacteria and phages, and these evolutionary trade-offs between phage resistance and antibiotic sensitivity do not depend on the same cell surface components, such as OMPs. Because T4P is a common receptor for phages (27, 28), the evolutionary trade-off we provided is likely the general mechanism. Collectively, we proposed that the T4P-mediated adsorption deficiency induced phage resistance and the changes in structure or composition of membranes are presumably the major cause for antibiotic sensitivity. The value of the findings reported here is that we reveal a complex trade-off mechanism between the phage and antibiotic. Only certain antibiotics, not all, after combined treatment with phages, may be effective. Our study will provide valuable information of phage-antibiotic combinations for treating MDR P. aeruginosa infections.

## MATERIALS AND METHODS

### Bacteria and phages.

The P. aeruginosa PAO1 strain (GenBank accession number NC_002516.2) from Shandong university, China, and its resistant mutant PAO1-R1 (accession number GWHBJTZ00000000) that we isolated were cultured in Luria-Bertani (LB) broth at 37°C and stored in 20% glycerol at –80°C until use.

P. aeruginosa phages vB_Pae_QDWS and vB_Pae_W3 were isolated against the host bacterial strain P. aeruginosa PAO1, from water samples collected from Qingdao, China. The phages were prepared using sodium chloride-magnesium sulfate (SM) buffer (10 mM MgSO_4_, 50 mM Tris-HCl, 100 mM NaCl, pH 7.5) supplemented with 20% glycerol and stored at –80°C. The phage suspension titers were determined by the double-layer agar method using LB as the culture medium. The plates were incubated at 37°C for 12 h and the number of lysis plaques were counted.

PAO1-R1 was isolated as follows: 3 μL of Pseudomonas phages mixture (10^5^ PFU/mL) were plated on PAO1 strain and cultured at 37°C for 60 h. Then the single-colony resistant strain was selected and purified by streaking. The acquired resistant strain was further verified by classic spot tests, as described (13).

### Transmission electron microscopy (TEM).

P. aeruginosa PAO1 and PAO1-R1 cells were grown in LB broth to an OD_600_ of 2.0, harvested, and washed in PBS. For phages, high titers (1 × 10^10^ PFU/mL) of phages were prepared for analysis. For adsorption analysis, high titers of phages were added to the P. aeruginosa cells with a multiplicity of infection (MOI) of 100. After 5 min of adsorption, the bacteria particles or phage pellet were deposited on carbon-coated copper grids and negatively stained with 2% phosphotungstic acid (pH 6.8). The samples were observed under a JEM-1200EX transmission electron microscope (JEOL) at 100 kV.

### Motility and biofilm production assays.

Swimming motility and twitching motility were assayed on agar plates containing specific medium according to the reported protocol with some modifications (14). For the swimming assay, strains were grown to an OD_600_ of 2.0. Next, 3 μL of the culture was spotted onto the semisolid medium consisting of 0.8% nutrient broth, 0.5% glucose, and 0.3% agarose. For twitching motility, the fresh colonies were stab-inoculated through the 1% LB agar layer. All the plates were inoculated at 37°C for 72 h. After incubation, the swimming motility zones were be observed. To measure the twitching motility zone, the agar was carefully removed, and the motility zone was measured by staining the Petri dish with 2% crystal violet for 1 h. Each strain was tested in quadruplicate at least.

Biofilm production was assayed on the wells of a 96-well microassay plate (15). Briefly, 200 μL of bacteria cultures were transferred into sterile 96-well plates. After incubation at 37°C for 18 h without shaking, cultures were gently removed, washed, and then stained with 0.1% crystal violet solution. To detach the biofilms, 200 μL of 30% glacial acetic acid was added into each well. The solubilized biofilm formations were finally measured at the wavelength of 570 nm by a multimode reader (Synergy LX, BioTek Instruments, Inc., Winooski).

### Adsorption rate assay.

PAO1 and PAO1-R1 strains were inoculated in LB medium until an OD_600_ of about 2.5 (about 6 × 10^9^ CFU/mL). Then the cells were diluted in LB and mixed with the phage vB_Pae_QDWS (3.25 × 10^5^ CFU/mL) and vB_Pae_W3 (1.37 × 10^5^ CFU/mL) solutions at an MOI of 0.001. The phage adsorption for vB_Pae_QDWS and vB_Pae_W3 were performed at 37°C for 5 min and 10 min, respectively. Finally, the cells were centrifuged at 10,000 rpm for 2 min at 4°C to obtain the free phages, which were detected using the double-layer agar method. The adsorption rate was calculated as follows: adsorption rate (%) = [(initial phage titer – phage titer in the supernatant)/(initial phage titer)] × 100 (16).

### Phage resistance assessment.

**(i) Spot test.** A total of 100 μL of the overnight cultures were mixed with 5 mL semisolid LB to form the double agar. The phage sensitivity of PAO1 and PAO1-R1 were detected by spotting 3 μL of the phage suspension with serial dilutions into the lawn of each strain. Then the samples were incubated at 37°C without shaking before examination.

**(ii) Lysis curves assay.** Lysis curves were generated following the addition of phage (MOI of 0.1) to a growing bacterial culture at an OD_600_ of 0.35. Then cultures were incubated at 37°C and shaken at 150 rpm with OD_600_ readings taken every 10 min on a multimode reader (Synergy LX, BioTek Instruments, Inc., Winooski).

### Outer membrane permeabilization assays.

The fluorescent probe 1-N-phenylnaphthylamine (NPN) was used to detect the outer membrane (OM) permeabilization activity. Briefly, PAO1 and PAO1-R1 were cultured in LB medium at 37°C with shaking at 200 rpm for 3 h. Cells were harvested by centrifugation, washed twice with 50 mM Tris-HCl buffer (pH = 7.5). The final cell suspension was adjusted to obtain an OD_600_ of 0.4. Next, 10 μM NPN were mixed with the P. aeruginosa cells, and the mixture was incubated in the dark at room temperature for 30 min. Cells treated with 10 μg/mL Polymyxin B were used as a positive control. Fluorescence, with an excitation wavelength of 350 nm and an emission wavelength of 420 nm, was recorded with a SynergyH1 microplate reader.

### Antibiotic susceptibility testing.

The antibiotic susceptibilities of PAO1 and PAO1-R1 were evaluated using a disk diffusion method (17). The overnight P. aeruginosa strains were subjected to a 1,000-fold dilution and mixed with the melted 2% agar LB medium. For antimicrobial susceptibility testing, the 6 mm diameter filter paper was sterilized and impregnated with 10 μL of antibiotic solution. Then, the paper was placed on P. aeruginosa lawns and the plates were incubated for 12 h at 37°C before detecting the inhibition halos. Tetracycline (10 mg/mL), moxalactam (10 mg/mL), cefsulodin (10 mg/mL), chloramphenicol (20 mg/mL), carbenicillin (20 mg/mL), streptomycin (50 mg/mL), gentamicin (10 mg/mL), polymyxin B (25 mg/mL, 6000 U/mg at 25°C), and kanamycin (50 mg/mL) were used in this assay.

MIC assays were determined by a 2-fold dilution technique in 96-well microtiter plates, as described by the Clinical and Laboratory Standards Institute (CLSI) guidelines. Briefly, the overnight P. aeruginosa PAO1 and PAO1-R1 strains were diluted 1:100 in Mueller-Hinton (MH) broth and grown at 37°C to OD_600_ = 1. Then, the cultured bacterial suspension was diluted to a final concentration of approximately 1 × 10^6^ CFU/mL. Various antibiotics with an original concentration of 1,024 μg/mL were used to make 2-fold dilutions. Then, 50 μL MH broth containing the antibiotics were mixed with 50 μL of bacterial cultures and added to a 96-well sterile microtiter plate. After incubation at 37°C for 18 h, the results were recorded. Measurements were performed in triplicate.

### Genome sequencing and bioinformatics analysis.

Genomic DNA was isolated using a Nucleic Acid purification kit (LC-Bio Ltd., Guangzhou, China) according to the manufacturers’ instructions. Whole genomes of strain PAO1 were sequenced using the Illumina MiSeq platform. The Velvet program was used to assemble the pair-end reads *de novo* with manually optimized settings. The genomes were submitted to the RAST web service for automated annotation followed by manual checking. The annotations are available via a guest account at the RAST website. Gene Ontology (GO) (http://geneontology.org/) and Kyoto Encyclopedia of Genes and Genomes (KEGG) (https://www.kegg.jp/) annotations were performed for the prediction of gene and protein functions.

### Data availability.

All data within the paper and the supplementary information file are available from the authors upon request. The complete genome sequence of phage vB_Pae_QDWS is available in GenBank under accession number MZ687409, and phage vB_Pae_W3 under accession number OK094665. The whole genome sequence data of PAO1-R1 reported in this paper have been deposited in the Genome Warehouse in National Genomics Data Center (45, 46), Beijing Institute of Genomics, and Chinese Academy of Sciences/China National Center for Bioinformation, under accession number GWHBJTZ00000000.
