# Supplementary material for: Phage Resistance Evolution Induces the Sensitivity of Specific Antibiotics in Pseudomonas aeruginosa PAO1
Source: Microbiol Spectr. 2022 Aug 16;10(5):e01356-22. doi: 10.1128/spectrum.01356-22 (PMC9603957; doi:10.1128/spectrum.01356-22)
Supplement: Supplemental file 1 — Supplemental material. Download spectrum.01356-22-s0001.pdf, PDF file, 0.4 MB [file spectrum.01356-22-s0001.pdf]

Supporting Information

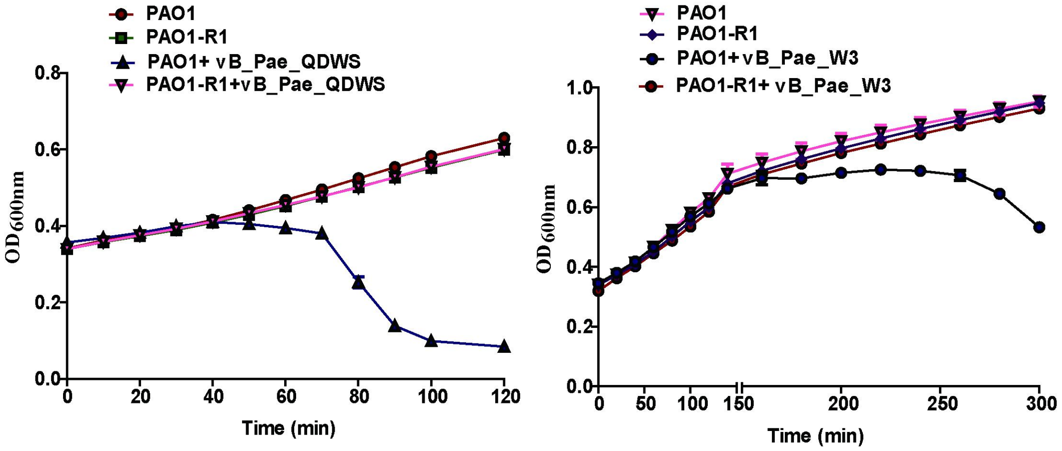

Fig. 1 Characterization of the phage-resistant strain PAO1-R1. Growth curves of PAO1 and PAO1-R1 infected with phage vB_Pae_QDWS or vB_Pae_W3 at an MOI of 0.1 in a 96-well microtiter plate containing 200 μL cultures were detected by a multi-mode reader. The values were the averages of three measures with standard deviation.

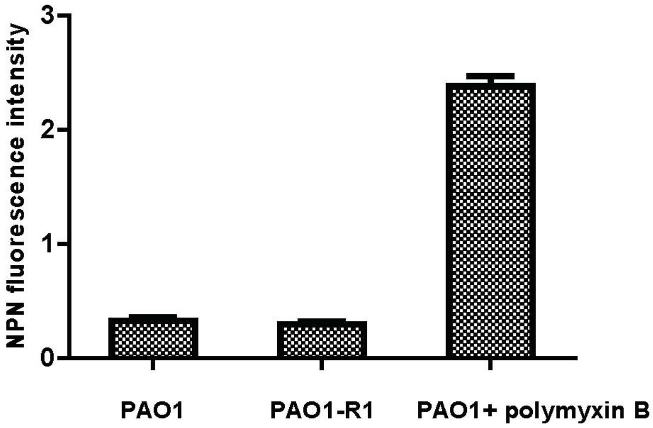

Fig. 2 Outer membrane permeabilization assays of PAO1 and PAO1-R1.

Table 1 SNPs found in PAO1-R1 using *P. aeruginosa* PAO1 as reference.

Gene name	Amino acid substitution	Product
PA0056	Ala230Pro	Probable transcriptional regulator
prlC	Gly405Ala	Oligopeptidase A
PA0116	Gly21Asp	HTH hxlR-type domain-containing protein
PA0159	Cys310Trp	Probable transcriptional regulator
pntB	Gln168*	NAD(P) transhydrogenase subunit beta
PA0202	Ala242Val	Probable amidase
mdcD	Ala246Thr	Malonate decarboxylase beta subunit
cysW	Gly21Ala	Sulfate transport protein CysW
glpQ	Ala24Pro	Glycerophosphoryl diester phosphodiesterase, periplasmic
PA0716	Ile45Thr	AAA domain-containing protein
lepA	Leu461Phe	Elongation factor 4
putA	Pro512Leu	Bifunctional protein PutA
PA0801	Gln108His	Uncharacterized protein
PA0839	Ala58Thr	Probable transcriptional regulator
PA0847	Ala376Val	Uncharacterized protein
phhC	Thr131Ile	Aromatic-amino-acid aminotransferase
aruE	Gly121Asp	Succinylglutamate desuccinylase
tolA	Ala241Thr	Tol-Pal system protein TolA
pyoS5	Ser343Leu	Pyocin S5
fliF	Gly156Asp	Flagellar M-ring protein
PA1108	Ala282Gly	Probable major facilitator superfamily (MFS) transporter
kefB	Lys393Asn	Glutathione-regulated potassium-efflux system protein KefB
PA1208	Gly477Ala	Uncharacterized protein
PA1245	Ala30Pro	AprX protein
PA1255	Pro30Ser	Probable trans-3-hydroxy-L-proline dehydratase
pmpM	Val268Met	Multidrug resistance protein PmpM
cheB1	Gly34Ala	glutamate methylesterase/protein-glutamine glutaminase 1
PA1611	Ala508Pro	Histidine kinase
pcrD	Gly489Ser	Type III secretory apparatus protein PcrD
cysS	Ala377Gly	Cysteine-tRNA ligase
PA1923	Ala295Gly	Putative Cobalamin biosynthesis protein CobN
PA1955	Ala92Gly	Uncharacterized protein
liuD	Ala368Gly	Methylcrotonyl-CoA carboxylase, alpha-subunit (Biotin-containing)

pslF	Ala42Pro	PslF, Glycosyltransferase
PA2271	Gly96Ala	Acetyltransferase
PA2375	Ala66Pro	Uncharacterized protein
PA2383	Ala158Gly	Probable transcriptional regulator
pvdJ	Pro819Ala	Amino acid adenylation domain-containing protein
PA2409	Ala16Pro	Probable permease of ABC transporter
mexT	Phe172Ile, Phe272Leu	Transcriptional regulator MexT
PA2503	Ala364Gly	Uncharacterized protein
PA2541	Ala165Gly	Probable CDP-alcohol phosphatidyltransferase
PA3173	Ala28Pro	Probable short-chain dehydrogenase
gltR	Ala234Gly	Two-component response regulator GltR
PA3259	Ser111Thr	Uncharacterized protein
PA3318	Ala16Pro	Uncharacterized protein
PA3455	Ala129Thr	Polyphosphate:AMP phosphotransferase
PA3522	Ala219Gly	Efflux pump membrane transporter
fruI	Ala893Gly	Phosphocarrier protein HPr
PA3760	His636Arg	Phosphoenolpyruvate--protein phosphotransferase
hpaA	Ala49Pro	4-hydroxyphenylacetate 3-monooxygenase oxygenase component
acoB	Ala81Pro	Acetoin catabolism protein AcoB
PA4179	Ala274Gly	Probable porin
pprA	Ala916Gly	Two-component sensor PprA
PA4341	Glu158Asp	Probable transcriptional regulator
PA4394	Val178Leu	Putative mechanosensitive ion channel family protein
proB	Ala225Gly	Glutamate 5-kinase
PA4968	Ala29Gly	Uncharacterized protein
pilQ	Lys693Glu, Val642Ile	Fimbrial assembly protein PilQ
PA5093	Ala173Gly	Probable histidine/phenylalanine ammonia-lyase
hutU	Thr431Arg	Urocanate hydratase
hutC	His42Pro	Histidine utilization repressor
phoU	Ala83Pro	Phosphate-specific transport system accessory protein PhoU
mtr	Lys286Asn	Aromatic amino acid permease

Table 2 Identified InDels in the genome of PAO1-R1 with the reference sequence of *P. aeruginosa* PAO1.

Genome Position	REF	ALT	Functional Annotations	Putative impact
169283	CG	C	PA0144<br>Putative nucleoside 2-deoxyribosyltransferase	Modifier
411125	AC	A	*fdx1*<br>Ferredoxin (4Fe-4S)	Modifier
667028	G	GC	PA0605<br>Probable permease of ABC transporter	Modifier
740419	G	GC	PA0683<br>Probable type II secretion system protein	High
816529	G	GC	PA0744<br>Probable enoyl-CoA hydratase/isomerase	Modifier
891099	A	AC	PA0809<br>Divalent metal cation transporter MntH 2	Modifier
1116213	G	GC	PA1026<br>Uncharacterized protein	Modifier
1215657	A	AG	PA1117<br>Uncharacterized protein	Modifier
1275766	GA	G	*napA*<br>Periplasmic nitrate reductase	High
1440622	CA	C	PA1327<br>Serine protease	High
1445357	A	AG	PA1328<br>Probable transcriptional regulator	Modifier
1467482	A	AG	PA1347<br>Probable transcriptional regulator	Modifier
1467483	C	CG	PA1347<br>Probable transcriptional regulator	Modifier
1835045	G	GC	*mtnC*<br>Enolase-phosphatase E1	High
1844903	T	TGTTGGC	*pscP*<br>Translocation protein in type III secretion	Moderate
2169348	A	AG	*agmR*<br>Glycerol metabolism activator	Modifier
2186927	G	GC	PA1995, Uncharacterized protein	Modifier
2195457	G	GC	*bdhA*<br>3-hydroxybutyrate dehydrogenase	Modifier
2239555	A	AG	PA2044	Modifier

			Putative cysteine protease	
2532046	G	GC	PA2296 Putative ABC transporter substrate-binding protein	Modifier
2753522	G	GC	PA2449 Transcriptional regulatory protein TyrR	Modifier
2807693	TCGGCCAGC	T	PA2492 Transcriptional regulator MexT	High
3016844	G	GC	PA2662 Uncharacterized protein	Modifier
3083196	A	AG	PA2729 Uncharacterized protein	Modifier
3777109	CAGGCCCCGG TCGCCATCGC CGG	C	*amiE* Aliphatic amidase	High
3919508	G	GC	PA3497 Uncharacterized protein	Modifier
4539468	G	GC	*ribH* 6,7-dimethyl-8-ribityllumazine synthase	Modifier
4888194	A	AG	PA4357 FeoC domain-containing protein	Modifier
5033101	G	GC	PA4492 DUF2135 domain-containing protein	Modifier
5071543	AACTG	A	PA4523 Uncharacterized protein	Modifier
5472415	C	CG	PA4870 DksA C4-type domain-containing protein	Modifier
5655220	C	CCGG	PA5024 Probable membrane transporter protein	Moderate
